# Excitatory versus inhibitory feedback in Bayesian formulations of scene construction

**DOI:** 10.1098/rsif.2018.0344

**Published:** 2019-05-01

**Authors:** Alireza Khatoon Abadi, Keyvan Yahya, Massoud Amini, Karl Friston, Dietmar Heinke

**Affiliations:** 1 Department of Mathematics, Faculty of Mathematical Sciences, Tarbiat Modares University, Tehran 14115-134, Iran; 2 Faculty of Informatics, Chemnitz University of Technology, Straße der Nationen 62, R. B216, 09111 Chemnitz, Germany; 3 Wellcome Trust Centre for Neuroimaging, Institute of Neurology, University College London, 12 Queen Square, London WC1N 3BG, UK; 4 Centre for Computational Neuroscience and Cognitive Robotics, School of Psychology, University of Birmingham, Edgbaston, Birmingham B15 2TT, UK

**Keywords:** selective visual attention, computational modelling, active inference, parallel distributed processing, neuroimaging

## Abstract

The selective attention for identification model (SAIM) is an established model of selective visual attention. SAIM implements translation-invariant object recognition, in scenes with multiple objects, using the parallel distributed processing (PDP) paradigm. Here, we show that SAIM can be formulated as Bayesian inference. Crucially, SAIM uses excitatory feedback to combine top-down information (i.e. object knowledge) with bottom-up sensory information. By contrast, predictive coding implementations of Bayesian inference use inhibitory feedback. By formulating SAIM as a predictive coding scheme, we created a new version of SAIM that uses inhibitory feedback. Simulation studies showed that both types of architectures can reproduce the response time costs induced by multiple objects—as found in visual search experiments. However, due to the different nature of the feedback, the two SAIM schemes make distinct predictions about the motifs of microcircuits mediating the effects of top-down afferents. We discuss empirical (neuroimaging) methods to test the predictions of the two inference architectures.

## 1. Introduction

In 2003, Heinke & Humphreys [1] introduced the selective attention for identification model (SAIM) to model translation-invariant object identification in multiple object scenes. A foundational assumption of SAIM is that the brain implements a soft constraint satisfaction as implemented by the parallel distributed processing (PDP) paradigm [2]. This led to a neural network architecture with feedback loops that enable an interaction between top-down information (i.e. knowledge about objects stored in an object identification stage) and bottom-up information (i.e. sensory information). Heinke and Humphreys demonstrated that SAIM could explain a broad range of empirical phenomena typically associated with selective visual attention, such as the effects of spatial cuing, object-based selection and the response time costs of recognizing multiple objects. Furthermore, SAIM could account for deficits in selective visual attention, such as visual neglect, visual extinction and the influence of knowledge on visual neglect.

In short, SAIM suggests that many ‘attentional’ phenomena can be explained as an emergent property of object identification (i.e. perceptual inference) in multiple object scenes. As far as we know, this level of success remains unrivalled by any other model. Subsequent work by Heinke and colleagues [3–5] demonstrated that extensions of SAIM could reproduce findings from visual search experiments, deal with natural colour images [6] and perceptual grouping [7]. Finally, by modifying the constraints to reflect action possibilities (i.e. affordances), it was possible to incorporate affordances in multiple object scenes [8]. It is also worth noting that SAIM's mechanisms are based on nonlinear dynamics that are formally similar to those used in dynamic neural fields (e.g. [9–13]). The latter reference is particularly relevant in the current context, because it considers the use of lateral interactions to engineer neurodynamic architectures for one-shot learning of visual objects using bottom-up recognition under top-down predictions. The common theme here is a dynamical implementation of a universal prior in object recognition; namely, that only one object (i.e. the winning or selected hypothesis) can cause sensory input at any one time. This fundamental prior is generally mediated by lateral interactions in neuronal schemes. The winner-take-all (WTA) interactions—implicit in SAIM—play the same role as lateral connections in neural field formulations.

The aim of this paper is to relate SAIM to a predictive processing framework for modelling action and perception; namely, the free-energy principle of Friston *et al*. (e.g. [14–17]; see [18,19]). A *prima facie* inspection suggests that Bayesian principles advocate a similar computational architecture to that employed by SAIM: both architectures are hierarchical, and both contain feedback loops. This paper offers a mathematical analysis of how these two architectures are related. In brief, we show that SAIM can be derived from first principles (i.e. the free-energy principle). However, SAIM assumes a different ‘generative model’ compared to those typically used in schemes like predictive coding. A crucial consequence of this difference is that SAIM's feedback loops are excitatory, while predictive coding schemes lead to inhibitory feedback loops (i.e. subtracting predictions from sensory input to form prediction errors). To facilitate a direct comparison between these two architectures, we derived a new version of SAIM—error prediction (EP)-SAIM—which uses the generative model usually adopted in predictive coding. We then present stimulation studies comparing the two models and produce (quantitative) predictions for future (EEG or fMRI) studies. In short, this work develops a formalism to address an important and long-standing systems neuroscience question: does the brain combine sensory information with prior knowledge using excitatory or inhibitory feedback?

To clarify the arguments, especially for those unfamiliar with SAIM, we first present a slightly revised version of SAIM. To highlight the contrasting assumptions about the feedback loops, we will call this version excitatory matching (EM)-SAIM. We then replicate a key finding from the foundational paper that introduced SAIM. Using simulations, we illustrate EM-SAIM's ability to perform object identification in multiple object scenes. Moreover, these simulations show that EM-SAIM reproduces the well-known multiple object cost; i.e. the increased time it takes to detect a target object with increasing numbers of non-target objects. This ubiquitous empirical finding is an emergent property of SAIM's WTA mechanism. The evidence for multiple object cost comes from visual search experiments (e.g. [20]; see [21] for a review). Here, we reproduce these results using the EM version of SAIM. Having established the validity of this EM scheme, we then reformulated the soft constraints in SAIM as free-energy minimization—to produce a prediction error (PE)-SAIM. We then repeated the simulation studies using the same (synthetic) stimuli to establish its construct validity, in relation to EM-SAIM. Finally, we compare and contrast the simulation results to identify key aspects of belief updating that may enable the two versions to be disambiguated, using empirical measures of neuronal evidence accumulation (e.g. EEG or fMRI). The MatLab code for the simulation studies reported in this paper can be found in the Github repository https://github.com/SAIM-models/EMvPE.

This paper does not aim to advance our understanding of selective visual attention *per se*; e.g. by comparing predictive coding and SAIM formulations of attention (e.g. [22,23]). Rather, we hope to lay the foundations for empirical work that will disambiguate between these convergent formulations (see General discussion). Finally, we have tried to keep the mathematics accessible for readers without a mathematical background.

## 2. The excitatory matching (EM)-SAIM

**Figure 1.**
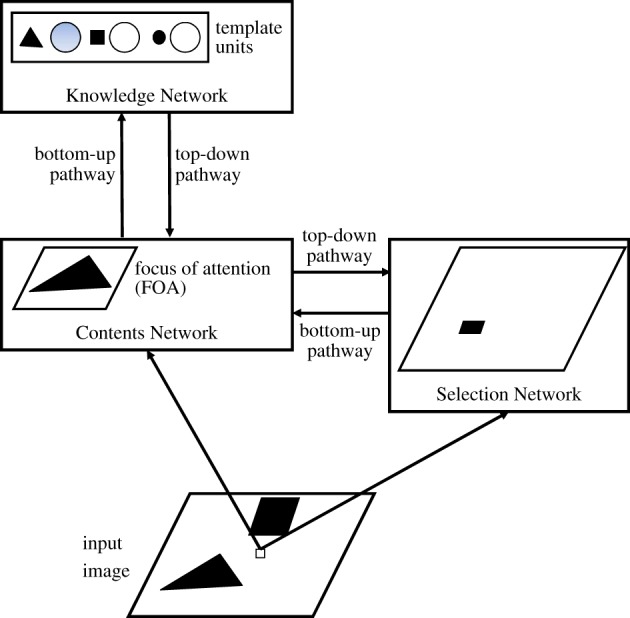
EM-SAIM's architecture. The three networks, Knowledge Network, Contents Network and Selection Network, have different functions: the Knowledge Network identifies the contents of the FOA by activating the best-matching template unit. The Contents Network maps a section of the input image into the FOA. The Selection Network determines the location of this section (see details in the main text). The arrows between the modules indicate the direction of message passing between the networks. (Online version in colour.)

Before presenting the mathematical derivation of EM-SAIM, we provide an overview of the EM-SAIM architecture (figure 1; for an illustration). After considering the mathematical details, we then highlight how an EM-SAIM differs from the original SAIM. We conclude this section by demonstrating that EM-SAIM can reproduce multiple object costs.

### 2.1. Overview

EM-SAIM selects an object by mapping a region in the input image into a ‘focus of attention’ (FOA) (figure 1). The mapping is implemented through the *Contents Network* and is translation invariant. This means that no matter where an object appears in the input scene, it can be mapped into the FOA. The *Selection Network* determines which region in the input image is mapped into the FOA. The *Selection Network* identifies this region by activating units in a layer that corresponds to locations in the input image (figure 1). The output of the *Contents Network* is passed onto the *Knowledge Network*. The *Knowledge Network* is equipped with template units that store templates of known (i.e. recognizable) objects. This network compares the templates and the input from the *Contents Network* with a simple template matching. Given the results of this template matching, the *Knowledge Network* activates the best-matching template unit. This reflects the identity of the selected object—the object in the *Contents Network*.

In addition to these bottom-up pathways, EM-SAIM also possesses top-down pathways. Note these top-down pathways are mandated by the soft constraint satisfaction approach described below. The top-down pathway from the *Knowledge Network* to the *Contents Network* adds a weighted sum of the templates to the activation in the FOA (excitatory feedback). The weighting is determined by the activation of the template units. In other words, the feedback directs the FOA to focus on the content of the *Contents Network*. The top-down connections from the *Contents Network* to the *Selection Network* underwrite a correlation of the *Contents Network* with the input image. The result of the correlation is feed into the *Selection Network*. Again—as with the feedback from *Knowledge Network *to* Contents Network—*this correlation rests on excitatory feedback. Since the *Selection Network* implements a WTA mechanism, this input directs the *Selection Network's* attention to the location in the input image that best matches the content of the *Contents Network*.

It is important to note that EM-SAIM does not achieve object identification instantaneously. Rather, object identification evolves over time. Initially (if we assume that there is no foreknowledge about the objects in the scene), the template units have same activation; the *Contents Network* is set to an equally weighted summation of template units and the *Selection Network* has equal activation across all image locations (i.e. no spatial bias). Subsequently, EM-SAIM begins the selection process and identification process in parallel, eventually converging to a point attractor, in which no unit changes its activation. At that point, EM-SAIM is said to have selected and identified an object.

### 2.2. Mathematical derivation

Our implementation of EM-SAIM is based on the energy function minimization scheme introduced by Hopfield & Tank [24]. In this scheme, the desired outputs of a network are expressed in terms of constraints; e.g. template matching as a constraint on the object identification in the knowledge network. Network dynamics can then be expressed as a gradient descent on an energy function E(y) of the output activity y of the neurons. The energy function comprises a mixture of distinct energy functions, where the minimum of each component satisfies a particular constraint. This ensures the network dynamics implement a form of soft constraint satisfaction. The general form of EM-SAIM uses the gradient descent described by Hopfield & Tank [24]

2.1 $$\tau {\dot{x}}_{i}=-\frac{\partial E(y)}{\partial y_{i}}.$$

Here, ${\dot{x}}_{i}$ is the transmembrane potential of the *i*th neuron (or neural population), $y_{i}$ is their firing rate activation and *τ* is the membrane time constant. The activation and depolarization are linked through a well-known sigmoid (activation) function: $y_{i}=f\;(x_{i})=1/(1+e^{-m(x_{i}-s)})$.

To ensure a level of biological plausibility, SAIM's energy function includes an energy component for every neuron or unit

2.2 $$E^{\mathrm{mem}}(y)=\frac{1}{\tau }\overset{N}{\underset{i}{\sum }}\int_{0}^{y_{i}}\;f^{-1}(z_{i})dz_{i}\;.$$

The gradient descent on this term leads to neuronal dynamics that emulate a leaky postsynaptic membrane.^1^ Another energy component, that is central to SAIM, is the WTA energy function

2.3 $$E_{\mathrm{WTA}}(y)=\frac{a}{2}{\left(\left(\overset{N}{\underset{i}{\sum }}y_{i}\right)-1\right)}^{2}-b\underset{i}{\sum }(y_{i}\;I_{i}).$$

Here, $I_{i}$ are the inputs to the *i*th neuron or neuronal population. This WTA energy function produces competition among neurons, in which the neuron with the largest input becomes activated—to nearly one (i.e. the winning unit), while all remaining neurons tend to zero. The first term corresponds to the constraint that the sum of all neuronal activities is equal to one; while the second term (i.e. input term) implies the constraint that the response of the neuron with the greatest input is maximal. The addition of the two ensures a WTA behaviour, where *a* and *b* weight the two constraints; allowing either constraint to dominate. The ensuing WTA behaviour is a nice illustration of *soft* constraint satisfaction. This energy function is important for the *Knowledge Network*, where the best-matching template is indicated by the highest input—and for *Selection Network*, as we will see later. It is also important to note that a change of the sign of the input term turns the WTA into a loser-take-all where the neuron with the smallest input wins the competition. This mechanism is important for PE-SAIM.

To ensure that EM-SAIM satisfies all constraints imposed by its constituent networks, the energy functions for each network are combined to provide an objective function for the entire network

2.4 $$\begin{array}{rl} E^{\mathrm{total}}(Y^{\mathrm{SN}},X^{\mathrm{CN}},y^{\mathrm{KN}})=\; & E^{\mathrm{mem}}(Y^{\mathrm{SN}},X^{\mathrm{CN}},y^{\mathrm{KN}})+\;E^{\mathrm{SN}}(Y^{\mathrm{SN}})\\ & +E^{\mathrm{CN}}(X^{\mathrm{CN}},Y^{\mathrm{SN}})+\;E^{\mathrm{KN}}(y^{\mathrm{KN}})\;. \end{array}$$

In other words, each network implements a constraint that is specified in terms of its unique energy function, while every neuron tries to minimize the total energy function: $E^{\mathrm{total}}$. Here, $\;E^{\mathrm{SN}}$ is the energy function for the *Selection Network*, $E^{\mathrm{CN}}\;$ is the energy function for the *Contents Network* and $E^{\mathrm{KN}}$ is the energy function for the *Knowledge Network* (i.e. superscripts SN, CN and KN stand for *Selection Network*, *Contents Network* and *Knowledge Network,* respectively).

The arguments of the energy functions, $\;Y^{\mathrm{SN}}$ and $y^{\mathrm{KN}}$, are the outputs of the *Selection Network* and the *Knowledge Network*, respectively, and $X^{\mathrm{CN}}$ is the output of the *Contents Network*. The use of X here indicates that—in contrast with the *Knowledge Network* and the *Selection Network*—we drop the sigmoid function in the *Contents Network*. This follows because the *Contents Network* represents continuous valued sensory signals. Also note the use of matrix notation for the *Contents Network* and the *Selection Network* outputs, which are two-dimensional matrices. By contrast, the *Knowledge Network* output is a one-dimensional vector. In the following, we will consider each individual energy function and the constraints it satisfies in detail.

#### 2.2.1. Knowledge network

The Knowledge Network implements template-based object identification through a scalar product

2.5 $$x_{k}^{\mathrm{temp}}=\overset{M,M}{\underset{i,j}{\sum }}x_{ij}^{\mathrm{CN}}\;w_{ij}^{k}.$$

**Figure 2.**
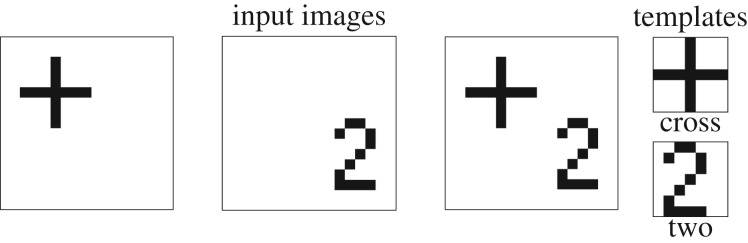
Input images and templates. The simulations used three input images and two templates in the Knowledge Network. The three input images were two single-object images (+ and 2) and one two-object image (+/2). The two templates perfectly matched the two objects.

Here, *M* is the size of the FOA and $w_{ij}^{k}$ is the template of the *k*th template neuron or unit. The size of each template is the same as the size of the FOA. Examples of templates can be found in the simulations below (figure 2). The Knowledge Network constraint ensures that the best-matching template unit is activated, while the remaining units are suppressed. The WTA energy function implements this constraint

2.6 $$E^{\mathrm{KN}}(y^{\mathrm{KN}})=\frac{a^{\mathrm{KN}}}{2}{\left(\left(\overset{K}{\underset{k}{\sum }}y_{k}^{\mathrm{KN}}\right)-1\right)}^{2}-b^{\mathrm{KN}}\overset{K}{\underset{k}{\sum }}y_{k}^{\mathrm{KN}}x_{k}^{\mathrm{temp}}.$$

#### 2.2.2. Contents network

The Contents Network receives an input from Sigma-pi units (i.e. modulatory synaptic interactions) which combine the activation in the selection network and the visual field to realize a translation-invariant mapping

2.7 $$I_{mn}^{\mathrm{CN}}=\overset{N,N}{\underset{ij}{\sum }}y_{i+m,j+n}^{\mathrm{SN}}\;y_{ij}^{\mathrm{VF}}.$$

Here, *N* is the size of the input image and $y_{kl}^{\mathrm{VF}}$ is the input image. Contents Network constraint ensures that the output units of the *Contents Network* reflect the output of the Sigma-pi units

2.8 $$\;E^{\mathrm{CN}}(X^{\mathrm{CN}},Y^{\mathrm{SN}})=-b^{\mathrm{CN}}\;\overset{M,M}{\underset{ij}{\sum }}\;x_{ij}^{\mathrm{CN}}\;I_{ij}^{\mathrm{CN}}.$$

#### 2.2.3. Selection network

The Selection Network implements one constraint, which ensures that only one location is selected. Here, we used the first term of the WTA energy function

2.9 $$E^{\mathrm{SN}}(Y^{\mathrm{SN}})=\frac{a^{\mathrm{SN}}}{2}{\left(\left(\overset{N,N}{\underset{lm}{\sum }}y_{lm}^{\mathrm{SN}}\right)-1\right)}^{2}.$$

This concludes our description of the network-specific energy components that constitute the total energy.

To simulate the processing of visual input, the total energy is minimized using a gradient descent scheme with the form of equation (2.1). In detail, we used an Euler approximation, with the addition of biological noise, of the sort implied by drift diffusion models (e.g. [25])

2.10 $$x_{i}(t)=x_{i}(t-1)-\frac{\partial E^{\mathrm{total}}(Y(t-1))}{\partial y_{i}}+\xi_{i}\;;\;\;\;\xi_{i}=N(0,\sigma ).$$

Here, $\xi_{i}$ is the noise term with variance σ. The resulting energy gradients for each network can then be expressed as follows (using direct calculation):

*Selection Network*

2.11 $$\begin{array}{rl} \frac{\partial E^{\mathrm{total}}(Y^{\mathrm{SN}},X^{\mathrm{CN}},y^{\mathrm{KN}})}{\partial y_{nm}^{\mathrm{SN}}}\;=\; & x_{nm}^{\mathrm{SN}}+a^{\mathrm{SN}}.\left(\left(\overset{N,N}{\underset{i,j}{\sum }}y_{ij}^{\mathrm{SN}}\right)-1\right)\\ & -b^{\mathrm{CN}}.\overset{M,M}{\underset{ij}{\sum }}x_{ij\;}^{CN}.y_{n-i,\;\;m-j}^{VF}\;. \end{array}$$

*Contents Network*

2.12 $$\frac{\partial E^{\mathrm{total}}(Y^{\mathrm{SN}},X^{\mathrm{CN}},y^{\mathrm{KN}})}{\partial x_{nm}^{\mathrm{CN}}}\;=x_{nm}^{\mathrm{CN}}\;-b^{\mathrm{CN}}.\;\overset{N,N}{\underset{i,j}{\sum }}y_{i+n,j+m}^{\mathrm{SN}}\;y_{ij}^{\mathrm{VF}}-b^{\mathrm{KN}}.\overset{K}{\underset{k}{\sum }}y_{k}^{KN}.w_{nm}^{k}.$$

*Knowledge Network*

2.13 $$\begin{array}{rl} \frac{\partial E^{\mathrm{total}}(Y^{\mathrm{SN}},X^{\mathrm{CN}},y^{\mathrm{KN}})}{\partial y_{k}^{\mathrm{KN}}}\;=\; & x_{k}^{\mathrm{KN}}+a^{\mathrm{KN}}.\left(\left(\overset{K}{\underset{i}{\sum }}y_{i}^{\mathrm{KN}}\right)-1\right)\\ & -b^{\mathrm{KN}}.\overset{M,M}{\underset{\;j,i}{\sum }}x_{ij}^{\mathrm{CN}}.w_{ij}^{k}. \end{array}$$

The terms in bold font (i.e. input terms in equation (2.3)) represent feedback from higher networks to lower networks; i.e. from the Knowledge Network to the Contents Network and from Contents Network to Selection Network. These terms follow from the gradient descent and show that feedback connections are required for soft constraint satisfaction. Crucially, these feedback connections constitute a positive (i.e. excitatory) feedback (see table 1 for the circuit diagram of the implicit message passing and connections). For example, responses in the Contents Network $x_{mn}^{\mathrm{CN}}\;\;$ will descend the gradient in equation (2.12), and will therefore increase with the activity of units in the higher Knowledge Network $y_{k}^{\mathrm{KN}}$. Similarly, unit responses in the Selection Network $y_{nm}^{\mathrm{SN}}\;\;$ increase with the source of descending projections from the Contents Network $x_{nm\;}^{\mathrm{CN}}$.

**Table 1.** Graphical illustration of feedback connections. These circuit diagrams illustrate how equations (2.11) and (2.12) for EM-SAIM and equations (3.3) and (3.4) for PE-SAIM map onto neural message passing and circuitry. Circles denote hypothetical neuronal populations, while the arrows correspond to connections. Excitatory connections are shown in black and inhibitory connections are shown in red. The small blue (crossed) circles denote a modulatory synaptic interaction (Sigma-pi units). These graphical illustrations illustrate why EM-SAIM can be seen as being mediated by excitatory feedback while PE-SAIM uses inhibitory feedback to implement a disinhibition via prediction error units. (Online version in colour.)

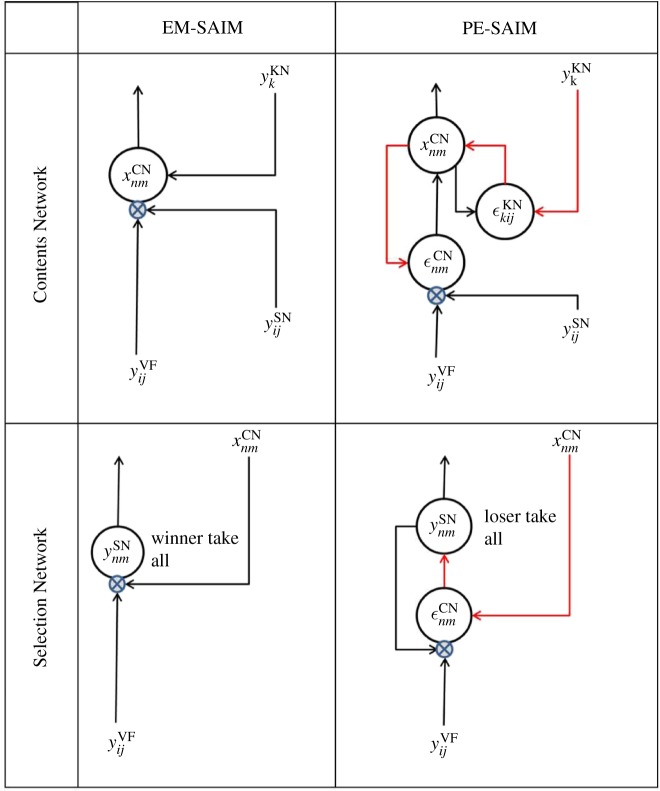

### 2.3. Comparing EM-SAIM with the original SAIM

EM-SAIM incorporates two changes that lend it a greater biological plausibility than the original implementation. The first is the inclusion of Brownian noise. This not only makes EM-SAIM more biological plausible but enables it to simulate variations in response time commonly found in behavioural experiments. The second change concerns the feedback connections. In the original SAIM, the feedback from the *Knowledge Network* was conveyed directly to the *Selection Network*. In EM-SAIM, the *Knowledge Network* now projects to the *Contents Network* and the *Contents Network* projects to the *Selection Network*. This change creates a more plausible architecture, given that feedback tends to target input brain region (e.g. [26]).

This revised feedback architecture retains the top-down modulation of the selection process, albeit in a more indirect way. To fully understand neurobiological premise of this argument, it is worth noting that SAIM's networks can be related to the *what*-pathway and the *where*-pathway (see [1] for a more detailed discussion). According to this interpretation, the *Knowledge Network* and the *Contents Network* correspond to brain regions in the *what*-pathway (ventral pathway), while the *Selection Network* corresponds to areas in the *where*-pathway (dorsal pathway), the posterior parietal cortex. Hence, if the *Knowledge Network* and the *Contents Network* are in the ventral pathway, feedback connections between these two networks better reflect known anatomical connections (as opposed to feedback connections to the *Selection Network* as in the original SAIM).

### 2.4. Simulation results

**Figure 3.**
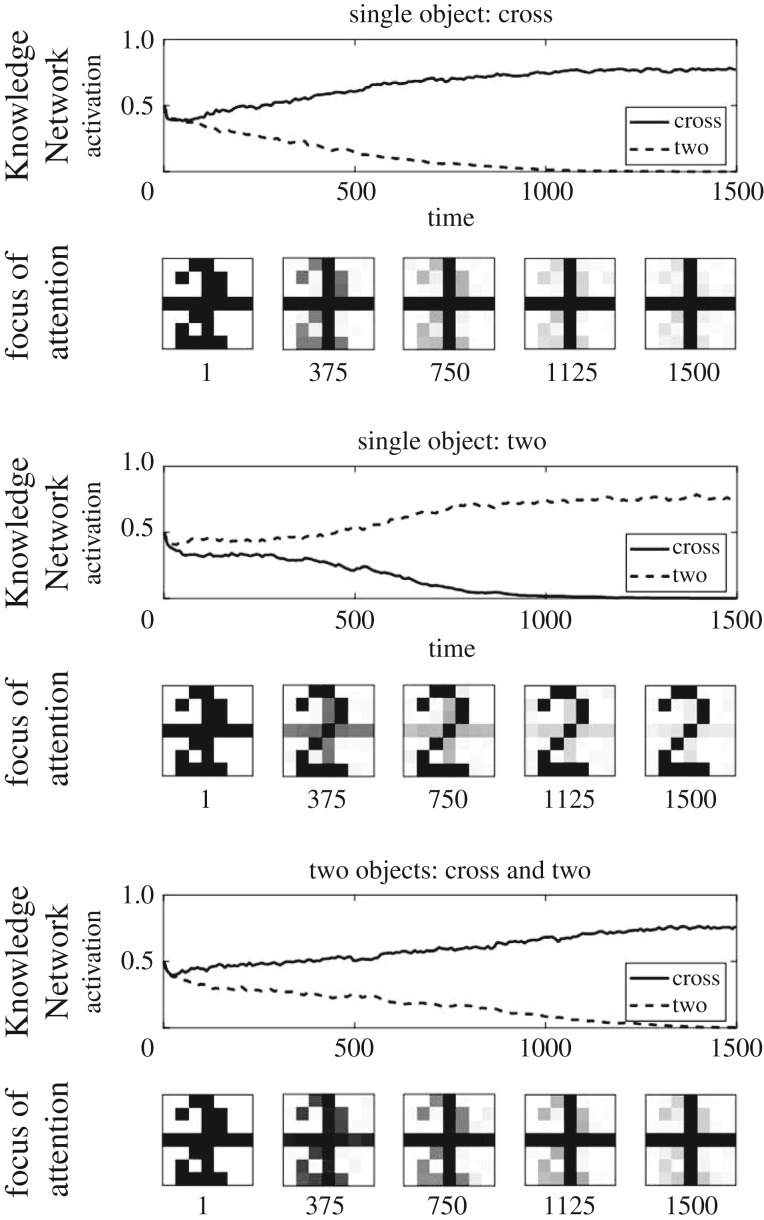
Three exemplar simulation results for multiple object costs with EM-SAIM. The graphs show the time course of the activation for the FOA and the two template units in the Knowledge Network. The reaction times were measured by determining the number of iterations it takes for a template unit to pass a threshold (0.9). As expected, the results show that EM-SAIM's reaction times were slower for the two-objects image (1013 iterations) than for the two single-object images: + (687 iterations) and 2 (777 iterations).

We first performed validation simulations to ensure EM-SAIM can replicate the simulations of multiple object cost in terms of reaction times, as reported in Study 2 of Heinke & Humphreys [1]. As in the original study, we used two objects, 2 and + (cross) (figure 2). These objects also formed the templates in the *Knowledge Network*. The reaction times were simulated by measuring the number of time steps it takes for a template unit to pass a threshold (see appendix A for parameters). The multiple object cost was simulated by contrasting the reaction times for input images with one object (+ or 2) with input images with two objects, + and 2. In empirical experiments, such as visual search tasks, multiple object costs are demonstrated with more objects (e.g. [20]; see [3] for a simulation study). However, for the purpose of this work, a simple set-up is sufficient to establish that EM-SAIM reproduces SAIM's cardinal behaviour. Figure 3 shows an example of a typical simulation for three input images: +/2, single 2 and single +.

**Figure 4.**
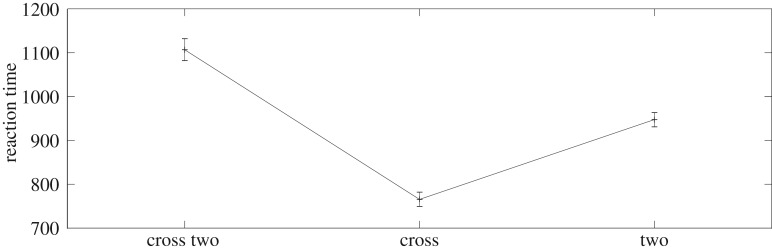
Results for 20 simulation runs for each input image. There was significant difference between +/2 and single +; and between +/2 and single 2. Hence, EM-SAIM can replicate the findings with the original SAIM (see main text for details).

These examples show that EM-SAIM can reproduce the multiple object cost. Also, as in the original SAIM, EM-SAIM exhibits a top-down bias towards the +, as the combined templates match better with the+than the 2. We also conducted a study with 20 simulations for each input image, to establish there was a statistically significant difference between the three conditions (figure 4). We applied a *t*-test to the simulation results and found a significant difference between +/2 and single + (*t*_38_ = 11.40; *p* < 0.001) and between +/2 and single 2 (*t*_38_ = 5.34; *p* < 0.001) (and between 2 and single + (*t*_38_ = −7.85; *p* < 0.001)). Crucially, the reaction time for +/2 was slower than for single + and single 2.

In summary, these simulation results suggest that EM-SAIM reproduces the key result from the original SAIM simulations. In addition to the original SAIM simulations, the new (EM) version can also reproduce the natural variation of reaction times found in experiments with humans. Also, despite the addition of neuronal noise, none of the 40 single stimuli simulations showed an error and the +/2 simulations always identified the cross. Note that the exact numerical outcome of the simulations, such as the variation of reaction times, depends on the parameter settings. Nevertheless, a broad range of parameter settings produce the findings present here. We will return to the issue of numerical evaluation of the model in the discussion section of PE-SAIM.

### 2.5. Interpreting selective attention for identification model within the active inference framework

In this section, we consider the links between the above formulation of visual processing within the PDP framework and current formulations based upon predictive coding and the Bayesian brain. In brief, we will see that both SAIM and approximate Bayesian inference can be described in terms of minimizing an energy function. The particular energy function used in Bayesian formulations corresponds to variational free energy (also known as an ‘evidence bound’ in machine learning). Variational free energy is a function of data and a generative model (i.e. a probabilistic model of how data are generated from causes, such as visual objects). In what follows, we show that the energy function used by SAIM can be interpreted as a variational free energy under a particular generative model. This means SAIM can be formulated in terms of Bayesian inference under a particular model of how visual data were generated. Furthermore, it means the computational architecture described in the previous section can be compared in a formal way to the architectures used in Bayesian schemes.

Casting SAIM in terms of variational free-energy minimization is much simpler than one might suppose. The free-energy principle considers how the Bayesian brain hypothesis (see [27] for a review) may be implemented in the brain. According to the free-energy principle (and in line with the Bayesian brain hypothesis), the brain is thought to use a generative model to infer the hidden (i.e. latent) causes of sensory signals. These models are characterized as ‘generative’ in the sense that they describe how the latent causes generate signals. In the course of the inference process, the brain is assumed to update representations (as encoded by a posterior probability density) of the latent causes via a minimization of ‘free energy’. This belief updating, evidence accumulation or inference process can be illustrated using SAIM's object identification.

Let us assume the generative model of object identification comprised the templates used in SAIM. Hence, for each physical object (e.g. two, crosses, etc.), the templates represent the latent causes of sensory signals in the input image. Given these sensory signals, the minimization of the free energy produces a posterior probability density for each template—reflecting the probability that the sensory signals are caused by the corresponding object. On this view, the templates correspond to prior beliefs about the latent causes of sensory signals that are recovered from sensory data through Bayesian belief updating. This belief updating can be expressed as a gradient descent on variational free energy.

An important point to note here is that the free energy minimized during inference is a single quantity (i.e. a functional of the posterior probability density and sensory input) that is specified by the generative model. In other words, the free energy is a global objective function analogous to SAIM's total energy function—and in both approaches, the energy has to be minimized. Hence, SAIM is, in effect, an instantiation of the free-energy principle. Moreover, a gradient descent on the free-energy functional implements the inference by optimizing the posterior distribution (e.g. [16]). In short, SAIM's gradient descent is formally consistent with the free-energy principle. In addition, one can regard SAIM's soft constraint satisfaction as equivalent to probabilistic inference under certain prior beliefs (i.e. constraints on the way visual data are generated).

Note that SAIM's inference process does not yield a representation of uncertainty, but simply a point estimate of the posterior. In Bayesian terms, this corresponds to a *maximum a posteriori* estimate. In terms of the free-energy principle, SAIM inverts a hierarchical Bayesian model, where the *Contents Network*, *Selection Network* and *Knowledge Network* encode the posterior expectations and hierarchical (also known as empirical) priors. Interestingly, the WTA constraints in SAIM can be regarded as implementing the prior belief that only one object can be in one place at a time.

Having noted a formal equivalence between SAIM's energy minimization approach and the free-energy principle, one can now ask: what is SAIM's underlying generative model? In the free-energy approach, the probabilistic generative model is linked and energy through a Gibbs measure

2.14 $$\ln p(Y^{\mathrm{VF}},\mu |m)=-E(Y^{\mathrm{VF}},\mu |m),$$

where $Y^{\mathrm{VF}}$ denotes sensory signals and μ are the expected causes of sensory signals under a generative model *m*. To reverse engineer the probabilistic representation in EM-SAIM, consider the energy function of EM-SAIM

2.15 $$\begin{array}{rl} \ln p(Y^{\mathrm{VF}},\;\;Y^{\mathrm{SN}},\;\;X^{\mathrm{CN}},\;\;y^{\mathrm{KN}})= & -E^{\mathrm{SN}}(Y^{\mathrm{SN}})-\;E^{\mathrm{CN}}(X^{\mathrm{CN}},Y^{\mathrm{SN}})\\ & -\;E^{\mathrm{KN}}(y^{\mathrm{KN}}). \end{array}$$

This equation can be separated into network-specific components, which correspond to the empirical and full priors of the generative model^2^

2.16 $$\begin{array}{rl} & p(Y^{\mathrm{VF}},Y^{\mathrm{SN}},X^{\mathrm{CN}},y^{\mathrm{KN}})\\ & \;=p(Y^{\mathrm{VF}},|Y^{\mathrm{SN}},X^{\mathrm{CN}})p(X^{\mathrm{CN}}|y^{\mathrm{KN}})p(Y^{\mathrm{SN}})p(y^{\mathrm{KN}}), \end{array}$$

with the likelihood and prior from the *Selection Network* becoming

2.17 $$\ln p(Y^{\mathrm{VF}},\;|X^{\mathrm{CN}},Y^{\mathrm{SN}})=\;b^{\mathrm{CN}}\;\overset{M,M}{\underset{mn}{\sum }}x_{mn}^{\mathrm{CN}}\;\overset{N,N}{\underset{ij}{\sum }}y_{i+m,j+n}^{\mathrm{SN}}\;y_{ij}^{\mathrm{VF}}$$

and

2.18 $$\ln p(Y^{\mathrm{SN}})=-\frac{a^{\mathrm{SN}}}{2}{\left(\left(\overset{N,N}{\underset{ij}{\sum }}y_{ij}^{\mathrm{SN}}\right)-1\right)}^{2},$$

and the empirical prior from the *Content Network* becoming

2.19 $$\ln p(X^{\mathrm{CN}}|y^{\mathrm{KN}})=b^{\mathrm{KN}}\overset{K}{\underset{k}{\sum }}y_{k}^{\mathrm{KN}}\overset{M,M}{\underset{ij}{\sum }}x_{ij}^{\mathrm{CN}}\;w_{ij\;\;}^{k}$$

and

2.20 $$\ln p(y^{\mathrm{KN}})=-\frac{a^{\mathrm{KN}}}{2}{\left(\left(\overset{K}{\underset{k}{\sum }}y_{k}^{\mathrm{KN}}\right)-1\right)}^{2},$$

where the prior from the *Knowledge Network* $p(y^{\mathrm{KN}})$ is a full prior.

These equations show that SAIM's generative model is formally distinct from those used in predictive coding, which uses Gaussian priors to ensure the priors are conjugate with the approximate (Gaussian) posterior (this is known as the Laplace assumption in Bayesian statistics). Under Gaussian assumptions, the likelihood and empirical priors above would have quadratic forms. However, it is immediately evident that the generative model implicit in SAIM has a much richer form. For example, the full priors in equations (2.18) and (2.20) show that EM-SAIM's model assumes a sparse probability density over the causes in the *Selection* and *Knowledge Networks*. This follows because these prior energies are minimized when one of the latent (non-negative) causes are one and the rest are zero. This sort of non-Gaussian prior is commonly employed in LASSO (least absolute shrinkage and selection operator) regression analyses (see Discussion). We will now look more closely at this form and elaborate a variant of SAIM whose empirical priors can be expressed in terms of squared prediction errors.

## 3. The PE-SAIM

In the previous section, we formulated SAIM in terms of free-energy minimization under a particular generative model that entails non-Gaussian empirical priors, in contrast with predictive coding models that usually assume Gaussian forms. In this section, we modify EM-SAIM by adopting Gaussian assumptions in the generative model (called PE-SAIM) and examine whether this new version can replicate the multiple object cost findings above. Under Gaussian assumptions, the free-energy components can be expressed as squared *prediction errors*. In SAIM, this applies to two levels: the *Contents Network*, which predicts the activation in the input image modulated by the *Selection Network* via Sigma-pi units

3.1 $$\ln p(Y^{\mathrm{VF}}|X^{\mathrm{CN}},Y^{\mathrm{SN}})=-\frac{b^{\mathrm{CN}}}{2}\overset{M,M}{\underset{nm}{\sum }}{\left(\epsilon_{nm}^{\mathrm{CN}}\right)}^{2}$$

$$\mathrm{and}\;\epsilon_{nm}^{\mathrm{CN}}=\overset{N,N}{\underset{ij}{\sum }}(y_{ij}^{\mathrm{VF}}y_{i+n,j+m}^{\mathrm{SN}})-\;x_{nm}^{\mathrm{CN}},$$

and the *Knowledge Network* which predicts the content of the FOA

3.2 $$\ln p(X^{\mathrm{CN}}|y^{\mathrm{KN}})=-\frac{b^{\mathrm{KN}}}{2}\overset{K,M,M}{\underset{kij}{\sum }}{\left(\epsilon_{kij}^{\mathrm{KN}}\right)}^{2}$$

$$\mathrm{and}\;\epsilon_{kij}^{\mathrm{KN}}=\;x_{ij}^{\mathrm{CN}}-\;y_{k}^{\mathrm{KN}}w_{ij}^{k}.$$

As noted earlier, the use of $x_{ij}^{\mathrm{CN}}$ (rather than $y_{ij}^{\mathrm{CN}}$) reflects the fact that the *Contents Network* uses a linear output function. Finally, note that in PE-SAIM, the two WTA priors (i.e. softmax) becomes a loser-take-all (i.e. softmin)—as the *Selection Network* and *Knowledge Network* need to select the best predictors; i.e. minimize prediction error. To minimize free energy, we again used an Euler scheme for gradient descent, retaining biological noise as in EM-SAIM. The requisite gradients for each network or hierarchical level can be derived by direct calculation from the above expressions:

*Selection Network*

3.3 $$\begin{array}{rl} \frac{\partial E^{\mathrm{total}}(Y^{\mathrm{VF}},Y^{\mathrm{SN}},X^{\mathrm{CN}},y^{\mathrm{KN}})}{\partial y_{nm}^{\mathrm{SN}}}\; & =x_{nm}^{\mathrm{SN}}+\;a^{\mathrm{SN}}\left(\left(\overset{N,N}{\underset{ij}{\sum }}y_{ij}^{\mathrm{SN}}\right)-1\right)\\ & \;+\;b^{\mathrm{CN}}\overset{M,M}{\underset{ij}{\sum }}\epsilon_{ij}^{CN}{\;y}_{n-i\;m-j}^{VF}. \end{array}$$

*Contents Network*

3.4 $$\frac{\partial E^{\mathrm{total}}(Y^{\mathrm{VF}},Y^{\mathrm{SN}},X^{\mathrm{CN}},y^{\mathrm{KN}})}{\partial x_{nm}^{\mathrm{CN}}}\;=x_{nm}^{\mathrm{CN}}\;-b^{\mathrm{CN}}\;\epsilon_{nm}^{\mathrm{CN}}+b^{\mathrm{KN}}\overset{K}{\underset{k}{\sum }}\epsilon_{knm}^{KN}.$$

*Knowledge Network*

3.5 $$\begin{array}{rl} \frac{\partial E^{\mathrm{total}}(Y^{\mathrm{VF}},Y^{\mathrm{SN}},X^{\mathrm{CN}},y^{\mathrm{KN}})}{\partial y_{k}^{\mathrm{KN}}} & =x_{k}^{\mathrm{KN}}+\;a^{\mathrm{KN}}\left(\left(\underset{i}{\sum }y_{i}^{\mathrm{KN}}\right)-1\right)\\ & \;+b^{\mathrm{KN}}\;\overset{N,N}{\underset{ij}{\sum }}(\epsilon_{kij}^{\mathrm{KN}})\;w_{ij}^{k}. \end{array}$$

These equations map onto a neural architecture as illustrated in table 1. The summaries of neuronal message passing in table 1 illustrate why EM-SAIM can be seen as being mediated by excitatory feedback, while PE-SAIM uses inhibitory feedback to implement a disinhibition via prediction error units. For example, the influence of $x_{nm}^{\mathrm{CN}}\;$ on $y_{nm}^{\mathrm{SN}}$ is mediated by two inhibitory connections (via $\epsilon_{nm}^{\mathrm{CN}}$); namely, an inhibition of inhibition. As in the equations for EM-SAIM, we used bold to indicate the feedback terms between networks. However, in contrast with EM-SAIM, the feedback terms are mediated by prediction errors (i.e. the ϵ terms in equations (3.1) and (3.2)) that implement an *inhibitory* (i.e. negative) influence of higher levels on the low levels. This inhibitory feedback is mandated by the formation of prediction errors. For example, the gradient descent implied by equation (3.4) means that units in the content network $x_{nm}^{\mathrm{CN}}$ increase when prediction errors $\epsilon_{knm}^{\mathrm{KN}}$ decrease. In short, by introducing prediction errors, we effectively reverse the sign of the coupling between successive levels in the hierarchy.

This architecture is consistent with generic predictive coding schemes, in which the prediction errors at any level in a predictive coding hierarchy are formed by subtracting predictions to create a prediction error or mismatch. Before considering the implications for neuronal message passing in the brain, we need to first establish the construct validity of the PE-SAIM in relation to the multiple object cost.

### 3.1. Simulation results and discussion

**Figure 5.**
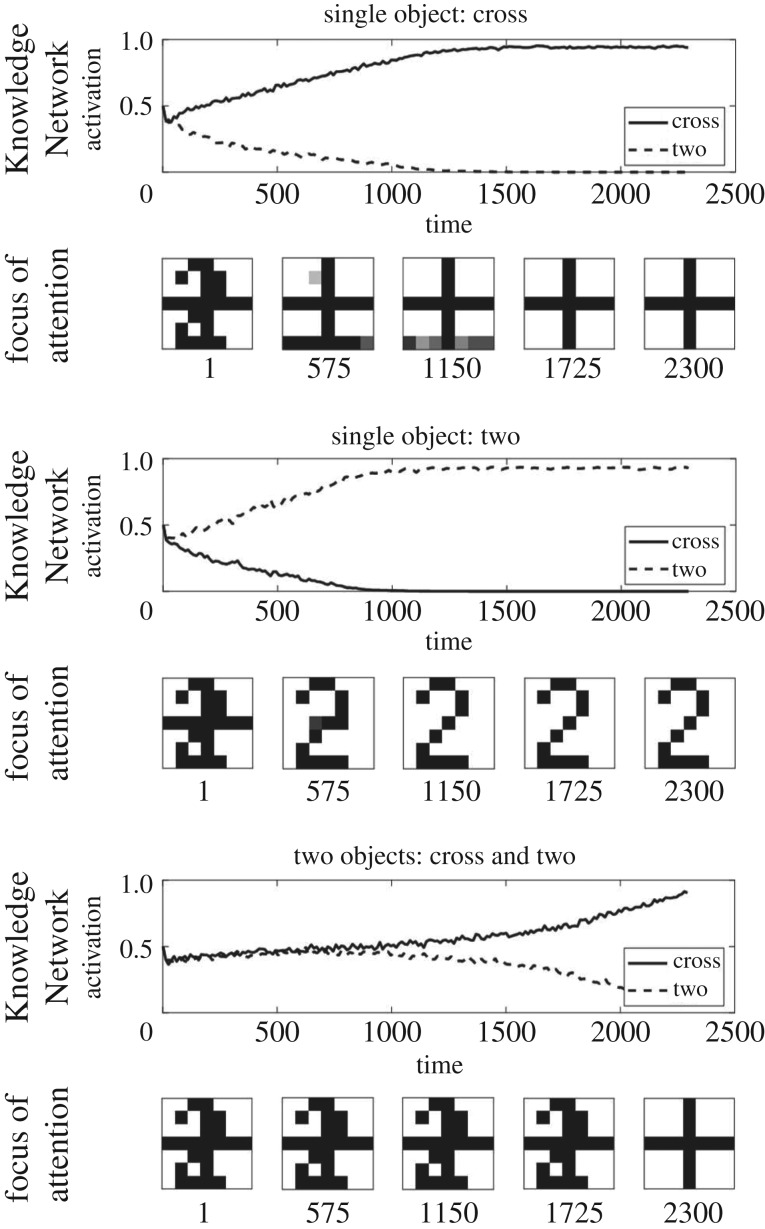
Three exemplar simulation results for the multiple object costs with PE-SAIM. The graphs show the time course of the activation for the FOA and the two template units in the Knowledge Network. The reaction times were measured by determining the number of iterations it takes for a template unit to pass a set threshold (0.56). As expected, the results show that PE-SAIM's reaction times were slower for the two-objects image (1159 iterations) than for the two single-object images:+(271 iterations) and 2 (267 iterations).

**Figure 6.**
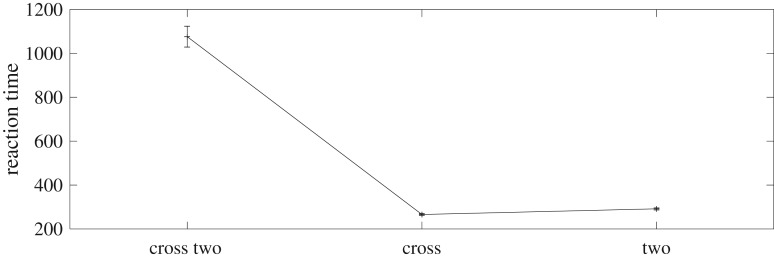
Simulation results for PE-SAIM from 20 runs for stimulus. There was significant difference between +/2 and single +; and between +/2 and single 2. Hence, PE-SAIM can produce the same results as EM-SAIM (see main text for details).

Figures 5 and 6 show simulation results that demonstrate PE-SAIM can also replicate the two-object cost. The *t*-test confirmed a significant difference between +/2 and single + (*t*_38_ = 17.09; *p* < 0.001) and between +/2 and single 2 (*t*_38_ = 16.52; *p* < 0.001) (and between 2 and single + (*t*_38_ = −4.00; *p* < 0.001)). Furthermore, none of the 40 single stimuli simulations showed an error and the +/2 simulations always selected the cross. Hence, both variants of SAIM can reproduce the qualitative multiple object costs. This is pleasing in the sense that it establishes a construct validity of the two schemes. In other words, both EM-SAIM and PE-SAIM can reproduce the finer (psychophysical) details of perceptual synthesis in recognizing multiple objects in visual scenes in a biologically plausible fashion. However, this presents an interesting challenge if we wanted to establish which offers the best account of neuronal message passing in real visual hierarchies. Recall from above that a key architectural difference between the two schemes is the use of top-down predictions to select the most likely explanation for sensory input in fundamentally different ways. The EM scheme uses *excitatory* feedback to ensure top-down constraints are satisfied in lower levels, while the PE scheme employs top-down predictions to form prediction errors using *inhibitory* feedback.

## 4. Comparing PE-SAIM with EM-SAIM

It is important to note that these particular simulation results depend on our particular choice of parameters.^3^ For both networks, the parameters were chosen to ensure significant reaction time cost effects in the absence of recognition errors. On the other hand, it would have been possible to generate simulation results where reaction costs are paired with recognition errors. Even though this observation is not crucial to make the point that, in principle, both models can replicate the two-object cost, it suggests the choice of parameters can modify the performance of object recognition in a measurable way. In turn, this affords the opportunity to compare the ability of the two schemes to explain empirical (e.g. psychophysical) data. This sort of comparison usually uses Bayesian model comparison. Bayesian model comparison has been used to disambiguate different models of choice behaviour and generally rests upon computing Bayes factors that score the evidence for one model over another, given the same data [28] (see [29] for a review). In brief, the Bayes factor assesses which model is better at generating a given dataset, considering all plausible parameter settings (under some generally uninformative prior over the parameters).

For the purpose of evaluating the two implementations of SAIM, Bayesian model comparison could leverage trade-offs between recognition accuracy and reaction time costs (similar to the effects observed in our simulations) by varying the number of objects and the discriminability of the stimuli. In this setting, it might be possible to use the two models to fit behavioural accuracy and response times, by optimizing model parameters. In principle, it would then be possible to compare the evidence for both schemes in empirical response data.

The simulations also illustrate an interesting point about the representation of the selected object in FOA. Despite the fact that there are no perfect representations of the selected object, both SAIMs can make correct decisions. This is the case because the ‘two’ can be easily discriminated from the ‘cross’. Note a perfect representation is not necessary as the task does not require it. Moreover, EM-SAIM's representation is less accurate than PE-SAIM's representation. This difference has the potential to distinguish between the two models. For instance, in an empirical study, participants could be required not only to find a certain object, but also to identify specific features of that object. Our simulations predict that inference under EM-SAIM would produce more errors than PE-SAIM. However, as noted above, this may depend the parameter settings, which would have to be optimized for any given choice behaviour, thereby enabling Bayesian model comparison to ascertain which model is the best account of empirical data.

Apart from these behavioural assessments, PE-SAIM and EM-SAIM can also generate neuronal responses of the sort measured by EEG or fMRI. Most current methods of measuring neuronal activity are indirect and depend on which physiological process (e.g. dendrite depolarization, axonal firing, haemodynamics, etc.) the respective method (EEG, fMRI, etc.) can measure. To simulate neuronal responses, we omitted the *Contents Network*—as its activation depends on ‘pixilated inputs’. We summed the output activation and the input activation (as defined by equations (2.11), (2.13), (3.3) and (3.5)) for the *Selection Network* and the *Knowledge Network*. We excluded the activation from the softmax/softmin equations in these calculations. The resulting neuronal response reflects activation in dendritic trees and axons, while ignoring activation of inhibitory interneurons.

**Figure 7.**
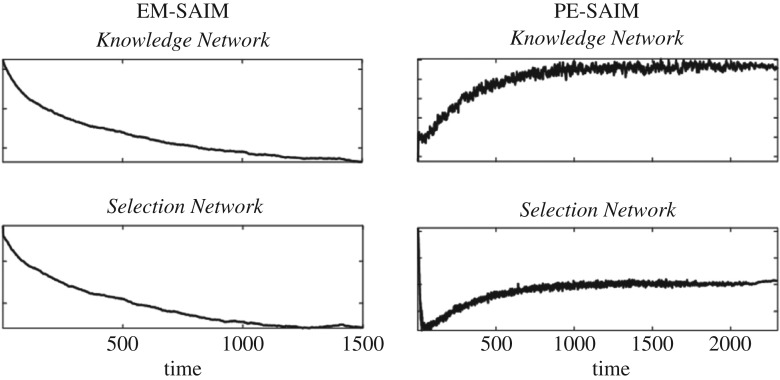
Sum of input and output activation. These results show that the two models predict a qualitatively different time course of neuronal activation (see main text for details).

Figure 7 shows the resulting time courses of activations for both models. They suggest that it may be possible to distinguish between the two models: for EM-SAIM, the results suggest a reduction in activity in both areas, while for PE-SAIM, they evince an increase. These results may come as a surprise for some readers: given that PE-SAIM tries to minimize prediction error, a reduction in activity might have been expected; while for EM-SAIM, the opposite effect might have been expected. The counterintuitive results with EM-SAIM can be explained relatively easily. The initial state of EM-SAIM uses a weighted combination of templates in the *Knowledge* *Network *and* Contents Network*. This combined template matches with the two objects in the input image (but the match is better for ‘cross’ than for ‘two’). As the selection process proceeds, this match declines as only the ‘cross’ in the input is matched—and the ‘two’ template in the *Knowledge Network* ceases to match. The increase in activation in PE-SAIM needs some more detailed unpacking. Initially, the combined template produces a top-down prediction that generates a better match for the ‘cross’ than the ‘two’. The *Selection Network* starts to bias the FOA towards the ‘cross’. Subsequently, this bias leads to a mismatch with the top-down prediction leading to an increased activation (i.e. prediction error). As the *Knowledge Network* starts generating the improved prediction—by selecting the cross—the increase in the prediction error declines in the input of the *Knowledge Network*. However, as the ‘two’ template produces a non-matching prediction, the overall error does not fall back to zero. A similar effect can be observed for the *Selection Network*. Even though the FOA generates a prediction matching the ‘cross’ in the input, the mismatch with the ‘two’ leads to higher activation. These results highlight the complicated nature of evoked responses when both prediction error and attentional selection are in play (see [30–32] for empirical examples in fMRI and EEG).

Other neuroimaging methods to exploit these sorts of simulations empirically could focus on disambiguating between excitatory and disinhibitory responses to top-down afferents. There are a number of candidates that one could consider. First, one could use the laminar specificity of forward and backward (bottom-up and top-down) connections in conjunction with laminar-specific fMRI to make predictions about the neuronal correlates of attentional effects [33]. Another approach would be to use frequency tagging to measure attentional effects on steady-state electrophysiological responses (e.g. [34]). There are also several examples in the literature that use *dynamic causal modelling* to disambiguate between inhibitory and excitatory connections in cortical hierarchies [35–41]. In brief, dynamic causal modelling entails fitting empirical (usually EEG—but see [42], for example, using fMRI) data—in the form of evoked responses—using a neural mass model with lamina-specific coupling [35,43]. One can then evaluate the evidence for competing architectures by specifying different patterns of connectivity within and between the neural masses that constitute electromagnetic sources (i.e. equivalent current dipoles). After the models have been fitted, the model evidence (i.e. the probability of the empirical data under each model) can be evaluated and used to adjudicate among different architectures. In principle, one could use exactly the same technology to test models that had different time constants—as well and different inhibitory or excitatory effects (e.g. [35]). This would involve comparing equivalent models with different priors over the synaptic time constants or effective connectivity in question (i.e. the influence of descending or feedback afferents to a primary visual source). In this setting, dynamic causal modelling will also have to consider that PE-SAIM assumes not only feedback loops between regions but also within layers (see the error terms in equations (3.4) and (3.5)). Recent invasive data, addressing the alternative architectures for predictive coding, also offer the intriguing possibility of testing the alternative predictions about the nature of feedback (see [44] for an example).

## 5. General discussion

The aim of the paper was to examine how SAIM's soft constraint satisfaction—using energy minimization—relates to the free-energy minimization of approximate Bayesian inference. To facilitate this comparison, we first created a new version of SAIM: EM-SAIM includes slightly more biologically plausible features than the original SAIM but crucially, for the purpose of this paper, is based on the same architecture and a formally similar energy function. We then ensured that EM-SAIM can reproduce the multiple object cost. Subsequently, we showed that SAIM's energy minimization can be interpreted in terms of Bayesian inference to a point estimator (i.e. maximum *a posteriori* estimate). We also noted that the ensuing probabilistic inference implements a soft constraint satisfaction, whereby empirical and full priors furnish the requisite constraints. By reverse engineering EM-SAIM's energy function, we showed that EM-SAIM's generative model uses a sparse prior of the sort commonly found in sparse regression models. It is worth noting that this type of prior is employed in methods such as the LASSO regression (e.g. [45]) and independent component analysis (e.g. [46]). The upshot of using this sort of prior is that it favours sparse representations of data. Furthermore, in EM-SAIM, the WTA forces the representation to become a local representation. Crucially, this generative model differs from the generative models used in predictive coding and related Bayesian filtering formulations of visual processing. These formulations normally employ a generative model based on Gaussian assumptions. Therefore, we replaced the empirical priors in EM-SAIM's architecture with a Gaussian form (i.e. log probabilities that are proportional to squared prediction errors) to show that PE-SAIM is also able to simulate the multiple object cost.

Our simulations suggest that EM-SAIM and PE-SAIM are quantitatively indistinguishable, in terms of their predictions of behavioural (psychophysical) responses. However, with suitable experimental designs, the two models can be used to model empirical data quantitatively. If this is feasible, Bayesian model comparison should be able to disambiguate the two schemes using recognition accuracy and reaction times (e.g. [28,29]). We further observed that EM-SAIM and PE-SAIM make quite different predictions about neuronal responses in terms of belief updating. EM-SAIM suggests that excitatory feedback loops mediate the behavioural effects we have illustrated, while PE-SAIM implies inhibitory feedback loops. Hence, these models seem to make distinct predictions about the physiology of feedback connections.

At first glance, EM-SAIM appears to be more consistent with the well-known physiology of excitatory (glutamatergic) feedback connections in the cortex (e.g. [47]). However, these feedback connections target inhibitory interneurons. Hence, it is possible that feedback connections can also mediate the construction of prediction error (see [16,43,48,49] for detailed arguments). Therefore, our current knowledge of physiology does not definitively disambiguate the two architectures. On the other hand—and as discussed above—it may be possible to distinguish between the two architectures empirically; leveraging the fact that the two models make different predictions for excitatory or inhibitory nature of top-down afferents. The two types of feedback motifs may generate different dynamics (with different time constants). It is therefore conceivable that laminar-specific fMRI, dynamic causal modelling or frequency-tagged EEG, in conjunction with Bayesian model comparison, might allow us to disambiguate the two architectures using non-invasive techniques in humans (see [50] for a contemporary discussion of empirical predictions for invasive studies). Finally, it is worth noting that both models make different predictions in terms of their preference for familiar versus novel stimuli.^4^ EM-SAIM would prefer familiar stimuli, while PE-SAIM would prefer novel stimuli (that elicit greater prediction errors). Interestingly, a recent study by Park *et al*. [51] found a category-specific (i.e. faces versus natural scenes) preference that could provide an interesting paradigm within which to test the two models.

The microcircuits for predictive coding motifs in table 1 speak to disinhibition as the physiological mechanism for the effect of descending or backward connections (indicated by the double red lines in table 1). There is growing interest and evidence for disinhibitory mechanisms of this sort (reviewed in [32,48,50]). This evidence comes in part from recent invasive studies using optogenetic characterizations of inhibitory interneurons. Microcircuit motifs that use disinhibition have been found in several cortical regions [52]: in brief, vasoactive intestinal peptide positive (VIP+) interneurons are thought to provide disinhibitory control, by targeting parvalbumin positive (PV+) and somatostatin positive (SOM+) interneurons that otherwise inhibit target excitatory neurons [53]. This synaptic architecture is supported by evidence from rodent studies, showing that optogenetic inhibition of SOM+ and PV+ interneurons reduces the inhibitory effect of descending projections to V1 from cingulate cortex. Conversely, optogenetic inhibition of VIP+ interneurons enhances the effect of projections from cingulate cortex [54]. In humans, disinhibitory effects can be observed when neocortical GABA is reduced using brain stimulation, both physiologically and functionally [55]. In short, the balance of empirical evidence points to the disinhibitory motifs that implied by a PE-SAIM like architecture.

The dialectic between excitatory and inhibitory feedback has been discussed in the literature at length (see [56–58]). For example, Kersten *et al.* [57] have formulated the dichotomy in terms of the ‘shut up’ versus ‘stop gossiping’ interpretations of Bayesian object perception. Intuitively, the shut up version corresponds to inhibitory top-down influences that ‘explain away’ any representations at lower levels to reduce the level of prediction error activity. Conversely, the suppression of activity in lower levels when something can be predicted may be better explained by top-down augmentation of the best representation that suppresses all competing expectations. Sometimes, the dichotomy is motivated by contrasting predictive coding with Grossberg adaptive resonance theory (ART) (e.g. [59]; see also Kay & Phillips's [60] coherence INFOMAX for a similar point; or Bowman *et al*.’s [61] salience detector). According to ART, the excitatory feedback loop is particularly important in the induction of strong ‘resonance’ to foster learning. Hence, the ART resembles EM-SAIM's architecture in terms of excitatory feedback.

Having established how SAIM is related to hierarchical Bayesian inference under the free-energy principle, it is worth returning to SAIM's domain of enquiry, modelling phenomena typically associated with selective visual attention. Predictive coding like formulations of attention introduce an additional variable that has to be optimized; namely, the amplitude of random fluctuations in sensory input—or its inverse called ‘precision’. This is a key quantity in engineering formulations of predictive coding (e.g. Kalman filtering). In this context, precision corresponds to the Kalman gain; namely, the gain or weight afforded prediction errors during belief updating. Crucially, the precision itself can be predicted. According to Feldman & Friston [22] and Kanai *et al*. [23], attention is realized as optimizing precision. In brief, top-down predictions of precision can select which prediction errors are effectively boosted, such that they have a greater influence on belief updating at higher levels of the hierarchy. This is thought to be the computational homologue of attention in predictive coding. Crucially, the top-down predictions of precision have an excitatory effect—in contrast with the inhibitory top-down feedback used to form prediction errors *per se*. When one considers predictions of precision, in the context of predictive coding formulations of attention, one has to consider both excitatory and inhibitory top-down feedback. Crucially, the excitatory top-down influences that mediate precision are modulatory or nonlinear in nature—in virtue of the fact that they modulate prediction errors. Interestingly, this speaks to the nonlinearities inherent in PE-SAIM.

In conclusion, attention is intricately linked with perceptual inference. Interestingly, this assumption is strikingly similar to the influence of SAIM's *Selection Network* using Sigma-pi units. Hence, it should be relatively straightforward to modify PE-SAIM and let the *Selection Network* modulate prediction error rather than the sensory information. We cannot foresee any problems in terms of functionality of this new PE-SAIM and anticipate it should behave in a similar way to the PE-SAIM described above. We will consider the formal relationship between precision and the role of the *Selection Network* in SAIM in a subsequent paper—and pursue the implications for the functional anatomy of visual attention.

## Endnotes

1 An intuitive explanation of this component is that its partial derivative ‘removes’ the integral leaving only the term $-f^{-1}(y_{i})$. The ensuing link between *x* and *y* turns this term into a leak term: $\left(-x_{i}\right)$.

2 Empirical priors are priors that are themselves parametrized by random variables. Empirical priors are part of any hierarchical generative model, with full priors at the highest level.

3 This is also true for the fact that EM-SAIM exhibits lower levels of noise than PE-SAIM.

4 We would like to thank the second reviewer for this idea.

## Data accessibility

The MatLab scripts for the publication can found on Github: https://github.com/saim-models/EMvPE.git.

## Appendix A. Parameter values

**Table:** EM-SAIM

network	parameter name	value
	threshold for reaction time	0.7
	maximal duration of simulation	1500
*Knowledge Network*	$\tau^{\mathrm{KN}}$	1000
	$a^{\mathrm{KN}}$	10
	$b^{\mathrm{KN}}$	0.1
	$s^{\mathrm{KN}}$	3.0
	$m^{\mathrm{KN}}$	30
	$\sigma^{\mathrm{KN}}$	6 × 10^−4^
*Contents Network*	$\tau^{\mathrm{CN}}$	600
	$b^{\mathrm{CN}}$	0.5
	$\sigma^{\mathrm{CN}}$	8 × 10^−4^
*Selection Network*	$\tau^{\mathrm{SN}}$	200
	$a^{\mathrm{SN}}$	15
	$s^{\mathrm{SN}}$	0
	$m^{\mathrm{SN}}$	5
	$\sigma^{\mathrm{SN}}$	0.0014

**Table:** PE-SAIM

network	parameter name	value
	threshold for reaction time	0.56
	maximal duration of simulation	2300
*Knowledge Network*	$\tau^{\mathrm{KN}}$	2000
	$a^{\mathrm{KN}}$	20
	$b^{\mathrm{KN}}$	1.5
	$s^{\mathrm{KN}}$	8
	$m^{\mathrm{KN}}$	50
	$\sigma^{\mathrm{KN}}$	7 × 10^−4^
*Contents Network*	$\tau^{\mathrm{CN}}$	500
	$b^{\mathrm{CN}}$	4
	$\sigma^{\mathrm{CN}}$	5 × 10^−4^
*Selection Network*	$\tau^{\mathrm{SN}}$	5000
	$a^{\mathrm{SN}}$	100
	$s^{\mathrm{SN}}$	5
	$m^{\mathrm{SN}}$	100
	$\sigma^{\mathrm{SN}}$	2.86 × 10^−4^
